# Use of organic material provided by an automatic enrichment device by weaner pigs and its influence on tail lesions

**DOI:** 10.1371/journal.pone.0309244

**Published:** 2024-11-01

**Authors:** Philipp Heseker, Jeanette Probst, Stefanie Ammer, Ulrich Hartmann, Mario Hasler, Nicole Kemper, Imke Traulsen

**Affiliations:** 1 Institute for Animal Hygiene, Animal Welfare and Farm Animal Behavior, University of Veterinary Medicine Hannover, Foundation, Hannover, Germany; 2 Department of Animal Sciences, Georg-August-University of Göttingen, Göttingen, Germany; 3 Chamber of Agriculture Lower Saxony, Bad Zwischenahn, Germany; 4 Applied Statistics, Christian-Albrechts-University of Kiel, Kiel, Germany; 5 Institute of Animal Breeding and Husbandry, Christian-Albrechts-University of Kiel, Kiel, Germany; University of Messina: Universita degli Studi di Messina, ITALY

## Abstract

Providing pigs with organic enrichment material is important for satisfying pigs’ natural explorative behavior to prevent injurious tail biting and thus increase animal welfare in general. The aim of this study was to investigate the effects of automatically supplied enrichment material of three different types (alfalfa pellets, oat bran pellets, or a mixture of both) and different enrichment frequencies (2, 4, or 6 supplies/day) on the behavior, the occurrence of tail biting, and daily weight gains of weaner pigs. The results showed significant effects and interactions of enrichment material, frequency and the time of day on the exploratory behavior, the occurrence of tail biting, and daily weight gains. Higher probabilities for pigs using the enrichment material were observed for groups provided with only two supplies/day or receiving oat bran pellets. Additionally, more pigs explored the material when supplied in the afternoon compared to the morning. Tail lesions began to increase in week 4 of the rearing period. Higher probabilities of having a tail lesion were recorded in groups provided with two supplies/day compared to four or six supplies per day. Furthermore, the highest probabilities for pigs having a tail length loss at the end of the rearing period were shown by groups receiving two supplies/day, with 0.170 for alfalfa pellets, 0.342 for mixture, and 0.486 for oat bran pellets. For daily weight gains, only alfalfa groups differed significantly from mixture groups in the case of two supplies/day. No differences were observed for the other factor combinations. These results showed the potential of an automatic enrichment device supporting pigs in performing their natural exploratory behavior in a conventional housing system. Higher numbers of daily enrichment supplies show beneficial effects to reduce the occurrence of tail biting and tail length losses.

## Introduction

Providing suitable enrichment material to pigs is very important to support the animals’ natural exploratory behavior and to prevent tail biting, one of the major challenges in pig farming [1, 2]. Tail biting leads to negative impacts on animal welfare, animal health, and growth rates, which also results in economic losses due to carcass condemnations at slaughter [3]. In nature, pigs have numerous opportunities for satisfying their need for exploration, like searching for feed by rooting, foraging, and sniffing [2]. This is restricted in barren environments where the lack of proper enrichment material is one of the main causes for the development of behavioral disorders like tail biting [4, 5]. This is additionally influenced by a variety of other risk factors, like genetics, availability and composition of feed and water, diseases, pen structure, space allowance, and the indoor climate [3]. The complexity of this multifactorial behavioral disorder makes predicting tail biting behavior very difficult [6]. Therefore, efficient prevention strategies are needed to avoid tail biting and increase animal health and welfare. The most common and effective, but strongly discussed strategy is to dock the pigs’ tail in the first week of life, although routinely shortening the tail is prohibited in accordance with the EU Council Directive 2008/120/EC and only allowed on farms with proven tail damage [7–11]. Tail docking itself causes acute pain and stress to the pigs and does not eliminate the underlying problem of tail biting as it only diminishes the symptoms [12]. The tips of docked tails show an uneven distribution of peripheral nerves with regressive changes and traumatic neuromas resulting in a higher sensitivity of the tail to pain than undocked tails, which explains the main effect for reducing serious tail biting [12, 13]. Nevertheless, tail docking is still very common on commercial farms in Europe with 95% of the tails being docked [14].

Many studies reported that mainly three different types of tail biting can be observed in pig husbandry. These are the two-stage tail biting, which is probably caused by a lack of proper enrichment material, the sudden forceful biting as a result of restricted feed or water access, and the obsessive tail biting that is carried out by single pigs which fanatically bite into other pigs’ tails [15, 16].

Moreover, offering organic enrichment material to the pigs in addition to the basic enrichment has become a very effective prevention strategy that gained a lot of attention in the last years. Combining the increase in space allowance per pig with the provision of straw as manipulable enrichment material for satisfying pigs’ exploratory behavior showed promising results as a method for prevention and intervention of harmful biting behavior [11, 17–24]. However, the use of long straw is controversially discussed due to plugging the manure system in fully slatted housing systems [25]. Therefore, Lahrmann et al. [26] compared the use of long and chopped straw, showing no differences in the straw length on any observed behavior or the occurrence of tail and ear lesions. Using processed materials like pellets has positives effects regarding the feed hygiene, as the materials are exposed to temperatures above 80°C during the pelleting process, thereby reducing bacterial count [27, 28]. Nonetheless, different types of non-straw enrichments can be provided to pigs for increasing exploratory behavior and reducing tail damage, either as a prevention or intervention measure [29]. Types of materials often including high fiber content like straw, peat, alfalfa, hay, corn, or silage were analyzed for their potential to occupy pigs and to reduce tail biting in the pens [24, 29, 30]. The materials are usually supplied once or twice a day during the routine animal check-ups and allocated in a feed dispenser, a rack, or a rooting tower in the middle of the pen [17, 20, 31]. First attempts to automatically supply organic enrichment material were made by Kauselmann et al. [32] who investigated the attractiveness of automatically filled material dispensers with chopped barley straw one or four times daily. No effect of the two refilling treatments on tail damage and tail length losses were observed. Nevertheless, the provision of straw and other loose enrichment materials is associated with higher labor costs [25, 33], so automatic enrichment devices may help farmers during their daily routines by regularly providing rooting materials to the pigs.

The aim of this study was to find out how different automatically supplied organic enrichment materials were used by weaner pigs and if the materials and the number of daily supplies have an impact on enrichment use, tail lesions, and daily weight gains.

## Materials and methods

### Animals and housing

The study was conducted at the experimental pig research farm of the Chamber of Agriculture Lower Saxony in Bad Zwischenahn in north-west Germany from February 2022 until January 2023. The research farm includes conventional housing systems for sows and suckling piglets, weaning and fattening pigs. Due to different research projects, the farm has several years of experience in housing pigs with intact tails. The animals were housed in accordance with EU (European Directive 2008/120/EC) and national law (‘German Order for the Protection of Production Animals used for Farming Purposes and other Animals Kept for the Production of Animal Products’ (TierSchNutztV, 2021)). The present study did not imply any invasive procedure or treatment to the animals and was reviewed and approved by the Animal Welfare Officer of the Chamber of Agriculture Lower Saxony in Oldenburg, Germany (approval number: A21-TS21923-LWK-3031-1).

For this study, a total of 840 weaner pigs (427 males and 413 females) were examined in six batches. Animals (Pietrain x (Landrace x Large White)) were born on the research farm, tails were kept undocked, the teeth were ground as needed and piglets received an antibiotic (Vetrimox® LA 150 mg/mL, 15 mg/kg, Ceva Tiergesundheit GmbH, Düsseldorf, Germany) on the 1^st^ day of life to prevent respiratory diseases. Between the 2^nd^ and 3^rd^ day of life, piglets were administered an iron injection (Forceris® 30/133 mg/mL, 200 mg/piglet, Ceva Tiergesundheit GmbH) to prevent iron deficiency anemia. Males were castrated within the first week of life (day 5–7) under isoflurane anesthesia (Isofluran CP® 1ml/mg, CP-Pharma Handelsgesellschaft, Burgdorf, Germany) accompanied by an analgesic (Metacam® 5mg/mL, 0.4 mg/kg, Böhringer Ingelheim Vetmedica GmbH, Ingelheim am Rhein, Germany) and an antibiotic (Vetrimox® LA 150 mg/mL, 15 mg/kg, Ceva Tiergesundheit GmbH) medication. Between the 5^th^ and 7^th^ day of life and 3–4 days before weaning, piglets were vaccinated against *Mycoplasma hyopneumoniae* and porcine circovirus (Porcilis® PCV M Hyo, 2 ml/piglet, Intervet Deutschland GmbH, Unterschleißheim, Germany). The piglets from the last batch of this study were additionally vaccinated against *Lawsonia intracellularis* (Porcilis® Lawsonia, 2 ml/piglet, Intervet Deutschland GmbH) before weaning. At weaning, pigs were on average (including the standard deviation) 26.9 ±1.5 days old, were weighed individually (mean weaning weight (including standard deviation) 7.4 ±1.3 kg) and received a colored ear tag for individual identification before being assigned and moved to the six pens in the weaning compartment. For this purpose, a maximum of three litters from the farrowing compartment were mixed to reduce stress and avoid fights after regrouping and only pigs with full-length tails and weighing more than 5 kg were selected. The weaner pens (2.5 x 5.5 m) housed 24 pigs each and consisted of one third iron slatted (dunging area) and two thirds plastic slatted flooring. Two drinking stations with nipple drinkers and a dry feeder (2.4 m length) were included in each pen. Pigs were fed *ad libitum* with three commercial diets and a change to the next diet was carried out gradually over five days. Basic enrichment was provided in the form of sisal ropes. Pigs were kept in the rearing compartment for 39 days before being individually weighed again for calculating the average daily weight gains (ADG) and then moved to the fattening compartment.

For enabling the provision of plant-based enrichment material, an automatic enrichment device (IBO Stalltechnik GmbH, Rhede, Germany) was installed in the weaning compartment. This consisted of two storage silos, a mixer with a scale, a feeding chain and dosing units for enabling pen-wise provision of pelleted enrichment materials (S1 File and S1 Fig). The material was released simultaneously in every pen, the pellets falling from the dosing unit through a pipe (1 m long) onto a plastic mat (0.6 x 1.2 m) placed on the floor where they became accessible for the pigs. Times of supplies, type and amount of material could be set on a feeding computer for each pen. Alfalfa pellets and oat bran pellets were used as enrichment materials separately or as a mixture of both. By using this automatic enrichment device, regular supplies of additional enrichment material per day were possible.

Furthermore, each pen was equipped with a video camera (AXIS 3206-LVE network camera, Axis Communications AB, Lund, Sweden), which continuously recorded the entire pen in top-down view with a resolution of 1,920 x 1,080 pixels and 20 frames per second. The cameras were attached to a network-attached storage (NAS-server; Synology DS720+, Taipeh, Taiwan) for storing the one-hour videos for further evaluations.

### Tail examinations

Every Monday and Thursday individual tail examinations in accordance with the ‘German Pig Scoring Key’ [34] were carried out by two trained observers. During the examinations, the observers entered the pen, identified the pigs by their colored ear tag and examined each tail carefully. Tail lesions were scored by using a four-point scoring system (Table 1) and tail length losses were recorded additionally (Score 0: no tail length loss; Score 1: tail length loss).

**Table 1:** Definition of the scoring system for tail lesions according to the ’German Pig Scoring Key’ [34].

Score	Definition
0	No visible lesion on the tail.
1	Superficial tail lesion (e.g., scratches).
2	Small tail lesions with a size smaller than the diameter of the tail.
3	Large tail lesions with a size larger than the diameter of the tail.

### Experimental design

Different settings were carried out by using the automatic enrichment device. In every batch, the six pens were randomly assigned to a material group (“AL” = alfalfa pellets, “OB” = oat bran pellets, “MI” = mixture of both) with two pens in a batch receiving the same material. The enrichment frequency differed between the batches (incomplete block design) and was set to two, four or six supplies per day (two replicates per number of supplies). The first three batches were randomly assigned to an enrichment frequency (4, 2, 6 supplies/day) and the order was then repeated for the next three batches. Times of supplies were distributed equally during the morning and afternoon between 08:00 h and 17:00 h. The amount of enrichment material per supply was set to 240 g/supply or 20/40/60 g/pig/day depending on the number of supplies per day.

### Behavioral observations

For determining the use of automatically supplied enrichment material, behavioral observations were carried out by using the OpenSource software BORIS (Behavioral Observation Research Interactive Software) [35]. Instantaneous scan sampling with a one-minute sampling interval was performed in the observation period from 5 minutes before the enrichment material was released from the dosing units until 15 minutes after providing the supply (21 scans in total). For each scan, every pig was assigned to a behavior according to an ethogram (Table 2) at pen level (number of pigs performing the specific behavior). Behavioral observations focused on one day per week (Tuesday) for the six weeks in the rearing compartment. Observations were stopped when farm staff entered the pen and behavior was affected. A total of 36 days, 807 supplies, and 16,896 scans were analyzed in this study.

**Table 2:** Ethogram for recording behavior traits using a one-minute scan sampling method.

Behavior	Description
**Active**	
Belly nosing	The pig is intensively manipulating another pigs’ belly with its nose.
Drinking	The pig is drinking in the modified drinking station.
Fighting	At least two pigs are fighting each other.
Mounting	The pig is jumping with its forelegs on another pig’s back.
Sisal rope	The pig is manipulating/exploring the sisal rope.
Standing	The pig is standing in the pen and not performing one of the other active behaviors.
**Enrichment**	The pig is manipulating/exploring/sniffing/rooting the automatically supplied enrichment material with its nose on the mat.
**Eating**	The pig is eating feed with its head in the feeding trough.
**Inactive**	
Lying	The pig is lying on its side or on its belly.
Sitting	The pig is sitting in the pen.

### Statistical analysis

The different behaviors belly nosing, drinking, fighting, mounting and sisal rope were summed up with the standing pigs as active pigs due to the low occurrence of those behaviors. Additionally, lying and sitting were combined as behaviors of inactive pigs. Statistical analysis was performed in R (Version 4.3.2) [36], including various packages such as lme4 [37], lsmeans [38], and gglot2 [39]. Due to the study’s focus on the effect of the treatment groups on the usage of the automatically supplied enrichment material, only this behavior was statistically analyzed, whereas the others were shown descriptively. A significance level of p<0.05 was used.

The effects of the treatment groups on the usage of the automatically supplied enrichment material were analyzed by using a generalized mixed-effects model [40]. Thereby, the number of pigs performing the specific behavior (enrichment) was set as the response variable following a binomial distribution (1 = performing the specific behavior, 0 = performing one of the other behaviors). Enrichment frequency (2, 4, 6 supplies/day), enrichment material (AL, MI, OB), and time of day (morning, afternoon) were included as qualitative fixed effects, whereas the rearing week (1–6) and the scan (6–20) were included as quantitative fixed effects (later for the consideration of significant differences, the corresponding means were used for the two quantitative factors scan and week). All interaction effects of the fixed factors were included in the model. The batch (1–6) and pen (1–6) were set as random factors in the models. The number of the scan was only taken into account in the model from scan 6 onwards, as the number of pigs assigned to the respective behaviors did not change in the previous scans, but changed very quickly as soon as the automatic enrichment device was triggered and the material became accessible for the animals. The logits were transformed from the binary response data to probabilities for visualizing the results.

For evaluating the impact of enrichment frequency and material on tail lesions and tail length losses, again generalized mixed effect models [40] were applied. For tail lesions, scores of the first two classes (score 0 and score 1) were summarized as “no lesion” and the other two classes (score 2 and score 3) were regarded as “tail lesion” for creating a binary response variable (0 = no tail lesion, 1 = tail lesion). For tail length losses, only the last scoring day at the end of the rearing period was used before the pigs were moved to the fattening compartment. Here, the tail length loss was set as the binary response variable (0 = no length loss, 1 = length loss). In both models, the enrichment frequency (2, 4, 6 supplies/day) and material (AL, MI, OB) were included as fixed effects. For the tail lesion model, the week was added as another fixed effect. All interaction effects of the fixed factors were included in the models. Batch (1–6) and pen (1–6) were included as random effects.

The effect of the enrichment frequency and enrichment material on the average daily weight gains (ADG) of the pigs (response variable) was analyzed by using a mixed model [41]. Enrichment material (AL, MI, OB), enrichment frequency (2, 4, 6 supplies/day) and their interaction were included as fixed effects, and batch (1–6) and pen (1–6) as random effects in the model. Normal distribution and homoscedasticity were checked with a graphical residual analysis. For all models, an analysis of variances was conducted [42], followed by multiple contrast tests [43, 44] in order to compare the several levels of supply, split for material and time of day and averaged over the week and the scan (or split for the weeks in the tail lesion model).

## Results

### Behavioral observation

**Fig 1:**
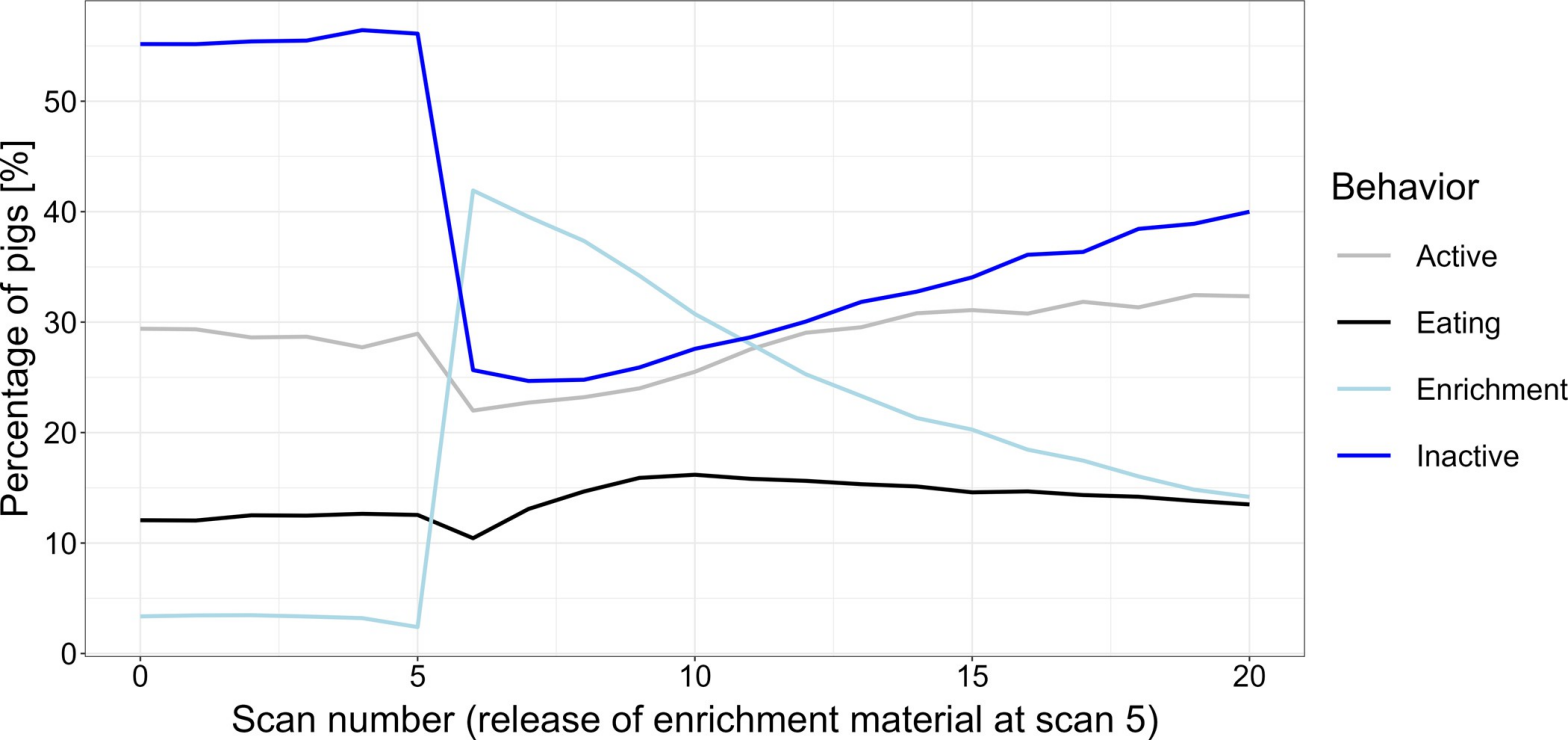
Average percentages of pigs performing specific behaviors in the observation period for all experimental settings. Average percentages of weaner pigs in a pen performing specific behaviors within an observation period from 5 minutes before until 15 minutes after the provision of enrichment material (at scan 5) averaged over the six weeks of the rearing period and all experimental settings during the six batches.

The observed animal behaviors changed within the observation period, especially as soon as the automatically supplied enrichment material became accessible for the pigs at scan 5 (Fig 1).

**Fig 2:**
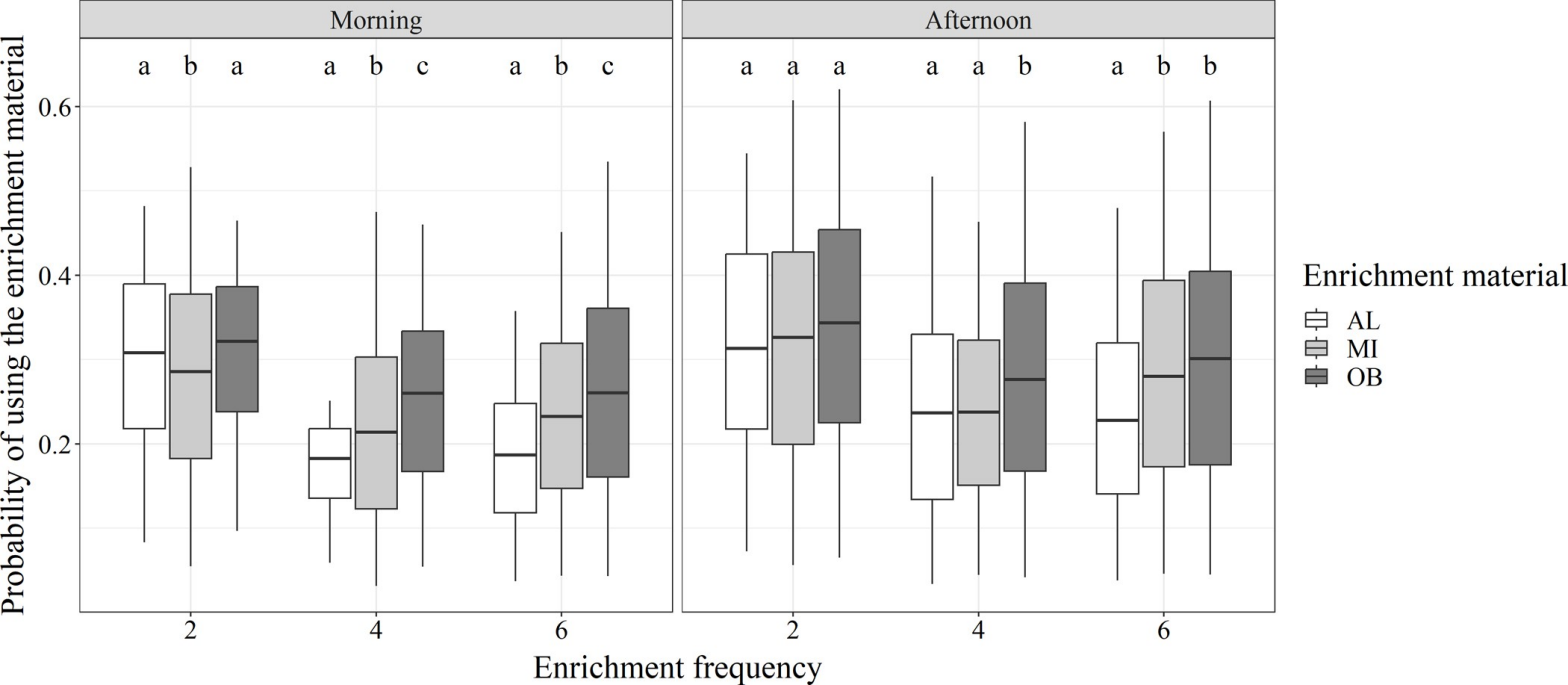
Effect of different enrichment materials on exploratory behavior of weaner pigs. Probabilities of pigs using different types of automatically supplied enrichment materials (AL = alfalfa pellets, MI = mixture of both, OB = oat bran pellets) for different times of day (morning, afternoon) and enrichment frequencies (2, 4 or 6 supplies/day). Significant differences are indicated by different letters (a, b, c). Boxplots were created by the probabilities for the included scans and weeks.

The results of the linear model showed that the probability of a pig exploring the automatically supplied enrichment material (S2 and S3 Files) differed significantly (p<0.05) for the fixed factors (enrichment material, enrichment frequency and time of day) in the model and showed significant interactions between them (for the mean week and mean scan of the observation period). Pigs use less the enrichment material as scan number increases and they get older. For comparing the materials, the supply of oat bran pellets showed the highest usage within several of the different parameter combinations of time of day and enrichment frequency and resulted in significant differences within the specific factor combinations (Fig 2).

**Fig 3:**
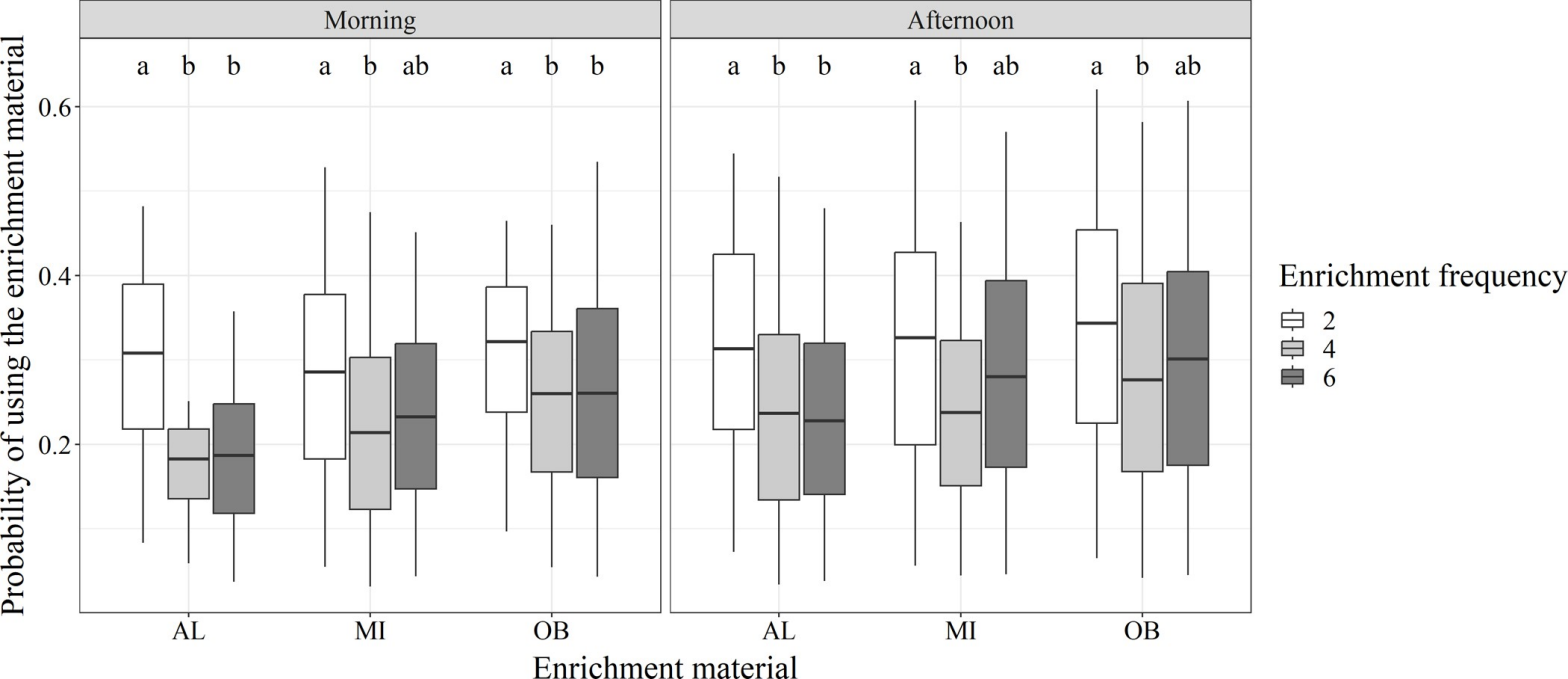
Effect of different enrichment frequencies on exploratory behavior of weaner pigs. Probabilities of pigs using enrichment material for different enrichment frequencies (2, 4, 6 supplies/day) for different times of day (morning, afternoon) and enrichment materials (AL = alfalfa pellets, MI = mixture of both, OB = oat bran pellets). Significant differences are indicated by different letters (a, b). Boxplots were created by the probabilities for the included scans and weeks.

For comparing the different enrichment frequencies (2, 4, 6 supplies/day), higher probabilities of a pig exploring the enrichment material were observed for groups that received two supplies per day compared to the groups with four or six daily supplies (Fig 3). Significant differences between the probabilities of the factor combinations (enrichment material and time of day) were presented in the model.

**Fig 4:**
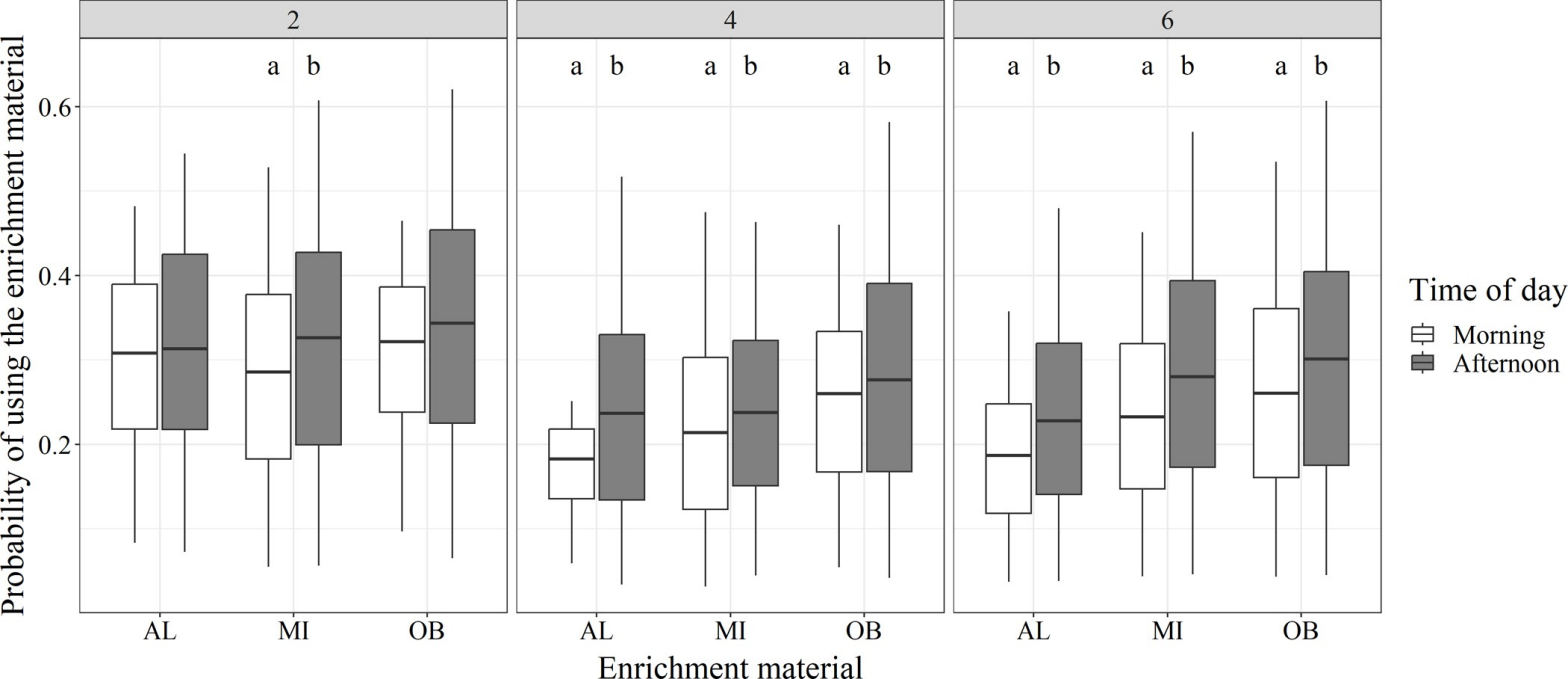
Effect of different times of day on usage of enrichment material by weaner pigs. Probabilities of pigs using the enrichment material for different times of day (morning and afternoon) for different enrichment materials (AL = alfalfa pellets, MI = mixture of both, OB = oat bran pellets) and enrichment frequencies (2, 4, 6 supplies/day). Significant differences are indicated by different letters (a, b). Boxplots were created by the probabilities for the included scans and weeks.

For the two factor levels of time of day (morning and afternoon), higher probabilities were shown for supplies that were offered in the afternoon than in the morning for most factor combinations of enrichment frequency and material (Fig 4).

### Effect on tail lesions and losses

Tail lesions began to increase in all groups in the second half of the rearing period with first significant differences occurring in the fourth week (Table 3). The occurrence of tail biting was significantly affected (p<0.05) by the enrichment frequency (2, 4, 6 supplies/day), the enrichment material (AL, MI, OB), the rearing week (1–6), and their interactions in the analyzed model (S4 and S5 Files). Highest probabilities of having a tail lesion (score 2 and 3) were observed for groups that received two automatic supplies of enrichment material per day (values above 0.5).

**Table 3:** Effect of different enrichment frequencies (2, 4, 6 supplies/day) and materials (AL = alfalfa pellets, MI = mixture of both, OB = oat bran pellets) on occurrence of tail lesions.

	AL			MI			OB		
	2	4	6	2	4	6	2	4	6
Week 1	0.000	0.020	0.007	0.009	0.014	0.006	0.012	0.008	0.002
Week 2	0.031	0.004	0.007	0.030	0.021	0.017	0.047	0.031	0.012
Week 3	0.049	0.020	0.007	0.030	0.017	0.023	0.052	0.046	0.015
Week 4	0.080	0.004	0.003	0.207^a^	0.021^b^	0.029^ab^	0.169	0.046	0.043
Week 5	0.359^a^	0.051^b^	0.194^ab^	0.418^a^	0.028^b^	0.291^a^	0.473	0.296	0.150
Week 6	0.526^a^	0.126^b^	0.210^ab^	0.602^a^	0.077^b^	0.296^ab^	0.701^a^	0.450^ab^	0.157^b^

^a,b^Different letters indicate significant differences (p<0.05) within the specific group.

A total of 80.5% of the pigs left the rearing compartment with full length tails at the end of the study. A significant interaction (p<0.05) for the enrichment frequency and material in the analyzed model (S6 and S7 Files) was observed (Table 4), but no significant differences (p>0.05) were found for groups with different enrichment frequencies. However, highest probabilities of a pig loosing part of the tail at the end of the rearing period were observed for groups with two supplies of enrichment material.

**Table 4:** Effect of different enrichment materials (AL = alfalfa pellets, MI = mixture of both, OB = oat bran pellets) and frequencies (2, 4, 6 supplies/day) on tail length losses.

AL			MI			OB		
2	4	6	2	4	6	2	4	6
0.170	0.001	0.118	0.342	0.004	0.028	0.486	0.123	0.032

### Effect on daily weight gains

**Fig 5:**
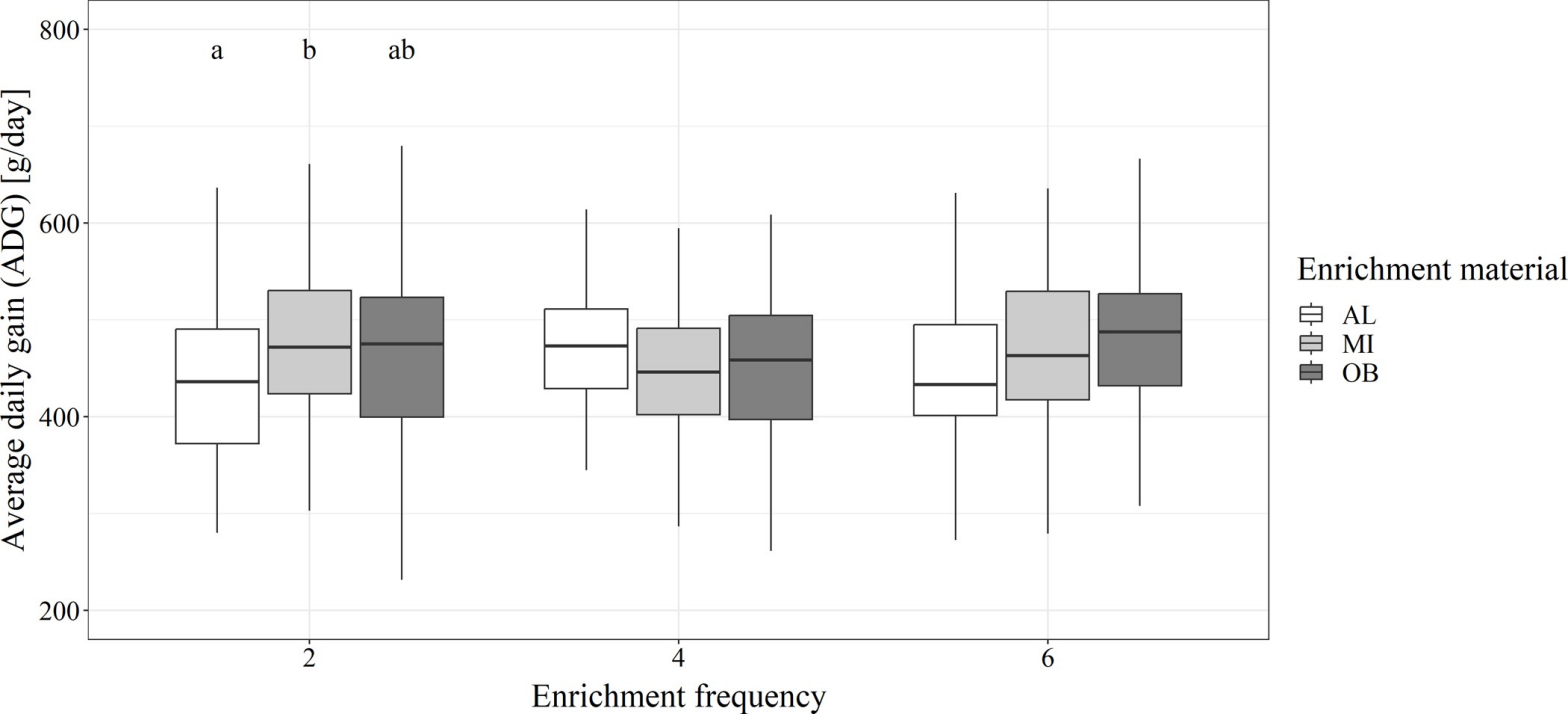
Effect of different enrichment materials on average daily weight gains of weaner pigs. Mean average daily weight gain (ADG) of weaner pigs in groups with different enrichment frequencies (2, 4 or 6 supplies/day) and materials (AL = alfalfa pellets, MI = mixture of both, OB = oat bran pellets) over the rearing period (39 days). Significant differences (p<0.05) within the frequency groups are indicated by different letters (a, b). The analyzed datasets can be found in the supplementary information (S10–S13 Files).

A significant effect (p<0.05) of the type of enrichment material on the ADG and a significant interaction (p<0.05) of enrichment frequency and enrichment material were found in the analyzed model (S8 and S9 Files). However, significant differences were only observed between the AL and the MI group when receiving the enrichment material twice daily (Fig 5).

## Discussion

### Number of pigs exploring the material

The probability of pigs exploring the automatic supplied enrichment material was significantly affected by the enrichment frequency (2, 4, 6 supplies/day), the enrichment material (AL, MI, OB), the time of day (morning, afternoon) and the interactions of the fixed effects. The probabilities of using the material decreased with increasing age of the pigs, which is in accordance with other studies [24, 30, 45]. Kauselmann et al. [45] investigated the exploration duration of flavored straw pellets by using UHF-RFID ear-tags and showed a decreasing exploration duration per pig and day during the rearing period. In their study, two peaks of exploration duration were observed throughout the day in the morning and afternoon with higher peaks during the afternoon hours, which is in accordance with our study, where higher probabilities for using the enrichment material were shown in the afternoon for several factor combinations. This is also in accordance with Ocepek et al. [30] who investigated higher exploratory behavior for supplies of different enrichment materials in the afternoon compared to the morning and in the time after the provision compared to the time before. Veit et al. [24] analyzed the effect of two enrichment materials on the number of occupied pigs and could not find a significant difference between the two treatment groups, but groups with alfalfa pellets tended to be less attractive than the corn silage. In our study, significant effects of the enrichment materials on the usage by weaner pigs were observed, but those effects were not consistent along all factor combinations (time of day and enrichment frequency). However, for groups with four or six supplies per day, higher probabilities for using the enrichment material were observed for the oat bran pellets compared to the alfalfa pellets. This might be explained by the pellet stability, which seemed to be better from the alfalfa, because the oat bran pellets broke apart much easier, which may result in a higher accessibility. Other approaches of automatically supplied enrichment material were carried out by Kauselmann et al. [32] who compared one and four automatic refillings per day in a rooting area of a material dispenser with chopped barley straw with regard to triggering additional exploratory or rooting behavior. However, the automatically supplied material was provided in addition to an *ad libitum* provision of chopped barley straw by a second dispenser (manually filled). In contrast to our study with higher probabilities of pigs exploring the material when receiving it only two times daily, no significant effect of the number of supplies per day on the exploration duration of the rearing pigs was observed in the aforementioned study. Additionally, a significant but not systematic effect of the rearing week on the exploration duration was found. Pigs’ exploratory behavior increased in all treatments after refilling the rooting area, which is in accordance with our findings [32]. The highest probabilities for foraging and using the enrichment material were observed one minute after the material became accessible. This attractiveness might be explained by the novelty of the material that is not permanently available in the pen and the pigs’ curiosity for discovering unfamiliar objects [5, 46]. If the pigs’ exploratory behavior is not stimulated, they redirect the behavior towards their pen mates [5]. Therefore, using manipulable materials became effective options as early intervention measures to reduce tail biting behavior when the material is not provided as a preventive measurement beforehand [46]. Intervention effects by an automatic enrichment device still need to be investigated. Limitations were, on the one hand, that the material was not accessible for every pig at the same time due to limited space on the plastic mat where the material was provided. This point is also influenced by the growth of the pigs and could lead to increased competition, restlessness, or aggression [24, 47], but was not investigated in this study. On the other hand, habituation to the material can be another possible explanation for the decreasing interest of the pigs regarding the exploratory behavior [45]. Therefore, different methods for the provision to enable free access to the enrichment material for all animals in the pen should be addressed in further studies.

### Evaluation of occurrence of observed tail lesions and losses

Tail biting is a multifactorial problem in pig husbandry that needs prevention measures other than routine tail docking [3]. The provision of loose enrichment material became one promising approach for the satisfaction of behavioral needs of docked and undocked pigs, reducing the occurrence of tail biting [1, 5, 11, 23, 31, 48, 49]. One commonly described type of tail biting is the two-stage tail biting that is caused by a lack of adequate enrichment material leading to increased oral manipulations of the pigs towards tails [15, 16]. Especially the use of straw on solid floor (e.g., twice daily) could significantly reduce the risk of pens showing bite marks and tail wounds compared to basic metal chains, rubber hoses, or straw provided in a rack [17]. In this study significant effects of the enrichment frequency and material on the occurrence of tail lesions were observed. More injurious tail lesions and losses were recorded for several groups that only received enrichment material twice a day compared to the groups with four or six supplies per day. Additionally, highest probabilities for having a tail lesions were observed for groups receiving oat bran pellets. This is partly in accordance with another study where the number of severe tail lesions was affected by the type of material but numbers of tail lesions were variable regarding the material and did not follow a consistent trend [24]. Additionally, the percentage of pigs having a tail loss varied from 9.9 to a maximum of 98.6% [24], which is much higher compared to this study where between 0.0 and 46.9% of the pigs in the observed batches lost part of their tail. A study of Studnitz et al. (2007) [5] stated that a positive aspect of providing extra enrichment materials to the pigs is the reduction of abnormal behavior towards penmates, which is in contrast to our study, where highest probabilities for using the material and for having a tail lesion were observed for groups receiving oat bran pellets. Kauselmann et al. [32] tested the effect of one and four automatic supplies of straw to weaner pigs and reported that 57.8% of the pigs had a full length tail at the end of the rearing period compared to on average 80.5% of the pigs having intact tails in this study. No effect of the number of refillings per day on the occurrence of tail damage was investigated in the aforementioned study. Tail biting was present in our study across all treatments groups, but highest probabilities for having tail lesions were observed for groups receiving two supplies per day [32]. This may be explained by the increased risk for the development of the two-stage tail biting because of restricting the pigs’ foraging behavior [15, 23]. Moreover, the provision of organic enrichment material not only serves as an effective mean to prevent tail biting, as it is also a very important tool for intervention of ongoing tail biting [17, 46]. Lahrmann et al. [46] reported significant effects of allocating extra enrichment material (straw on the floor or haylage in an elevated spherical cage) on tail biting outbreaks after the first damaged tail was recorded in a pen. In this study, only effects of prevention were investigated. Thus, further studies are needed to analyze the potential of automatically supplied enrichment materials for timely intervention of tail biting behavior.

### Effect on daily weight gain

In this study, significant effects of enrichment material and a significant interaction of enrichment frequency and material on the average daily weight gain were observed in the analyzed model. For groups receiving enrichment material twice daily, significant higher daily weight gains were observed for pigs receiving a mixture of alfalfa and oat bran pellets compared to pure alfalfa pellets. However, for the other enrichment frequencies, no significant differences were observed and therefore no clear explanation is found. Kalies et al. [31] reported 50g/day higher daily weight gains for fattening pigs that received straw from an interactive rooting tower compared to a control group. In contrast, Veit et al. [24] investigated similar weight gains for different raw materials (control, dried corn silage, alfalfa hay), which is in accordance with our study for four and six supplies per day. In a study by Holling et al. [20] the influence of providing straw from a foraging tower on the weight gains of rearing and fattening pigs compared to a control group without straw was investigated, but again, no significant difference between the different treatments were recorded. Lange et al. [50] showed higher daily weight gains for docked pigs compared to undocked pigs and explained this by the higher occurrence of tail biting within the group of undocked pigs and its negative influence on the performance of the pigs. This impact of tail lesions on the performance of pigs was not investigated in our study.

## Conclusion

This study showed a significant impact of the number of supplies and the type of organic enrichment material provided by an automatic enrichment device on the occurrence of tail lesions in weaner pigs. The highest probability of having a tail lesion was observed for groups that received the enrichment material only twice daily, although those groups showed highest usage of the supplied material. Therefore, it is recommended to offer the pigs more supplies daily to prevent tail lesions and losses without affecting the average daily weight gain. The usage of the automatically supplied material can be significantly affected by different factor combinations of enrichment frequency and material, but all in all this had positive effects on tail lesions and losses. Further studies are needed to evaluate more combinations of enrichment frequency and different materials, also in regard to creating targeted intervention strategies in case of the onset of tail biting behavior.

## Supporting information

S1 Fig Picture of the dosing unit of the automatic enrichment device in the pen. (JPG)

S1 File Schematic drawing of the automatic enrichment device in the compartment. (PDF)

S2 File Summary of the analyzed model on the usage of the enrichment material. (PDF)

S3 File Results of the analyzed model on the usage of the enrichment material. (PDF)

S4 File Summary of the analyzed model on the occurrence of tail lesions. (PDF)

S5 File Results of the analyzed model on the occurrence of tail lesions. (PDF)

S6 File Summary of the analyzed model on the occurrence of tail losses. (PDF)

S7 File Results of the analyzed model on the occurrence of tail losses. (PDF)

S8 File Summary of the analyzed model on the average daily weight gains. (PDF)

S9 File Results of the analyzed model on the average daily weight gains. (PDF)

S10 File Dataset of the usage of automatic supplied enrichment material. (XLSX)

S11 File Dataset of the occurrence of tail lesions. (XLSX)

S12 File Dataset of the occurrence of tail losses. (XLSX)

S13 File Dataset of the average daily weight gains. (XLSX)
