# Supplementary figures and images for: Use of organic material provided by an automatic enrichment device by weaner pigs and its influence on tail lesions
Source: PLoS One. 2024 Nov 1;19(11):e0309244. doi: 10.1371/journal.pone.0309244 (PMC11530003; doi:10.1371/journal.pone.0309244)

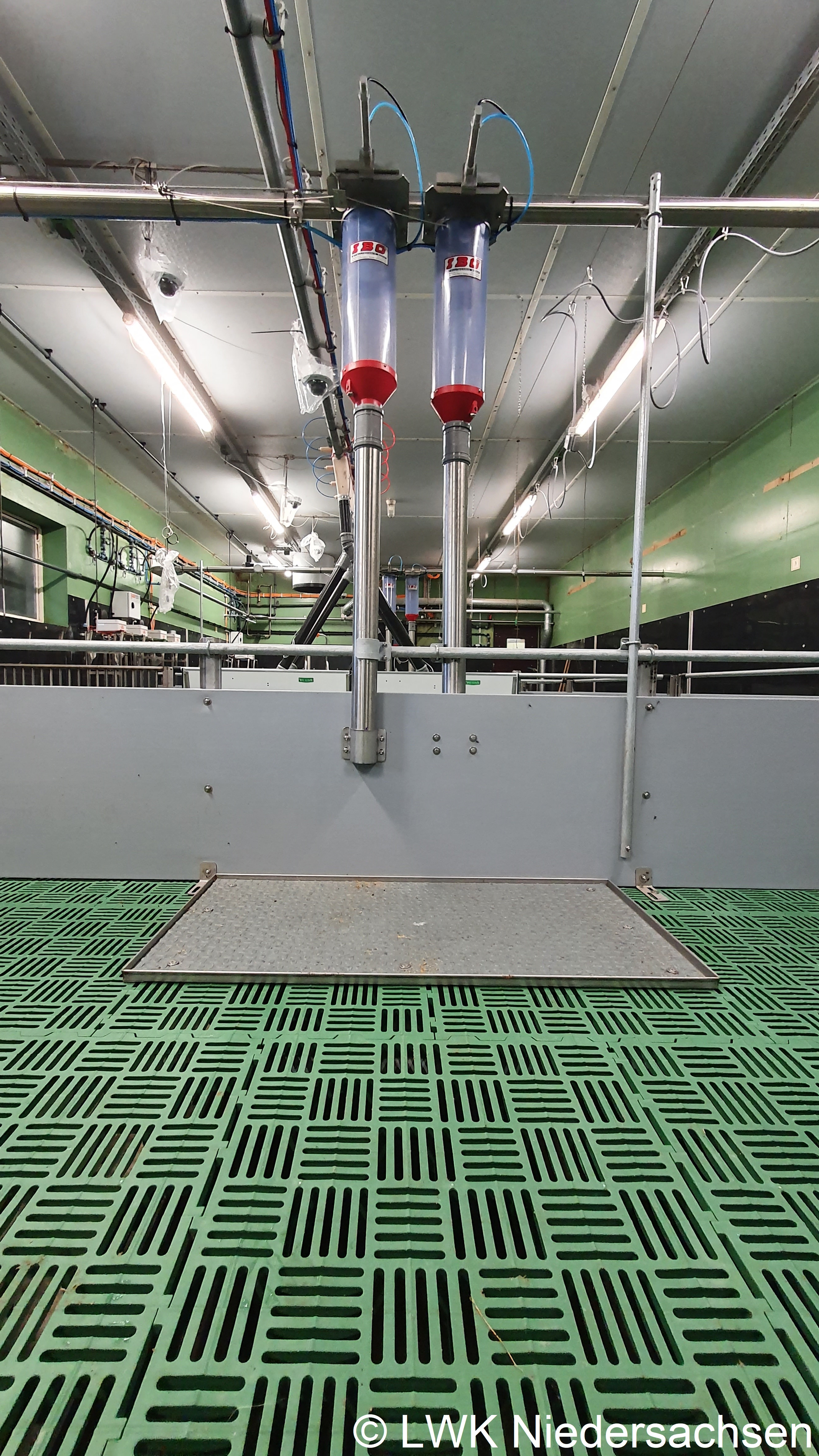

Supplement: S1 Fig — (JPG) [file pone.0309244.s001.jpg]

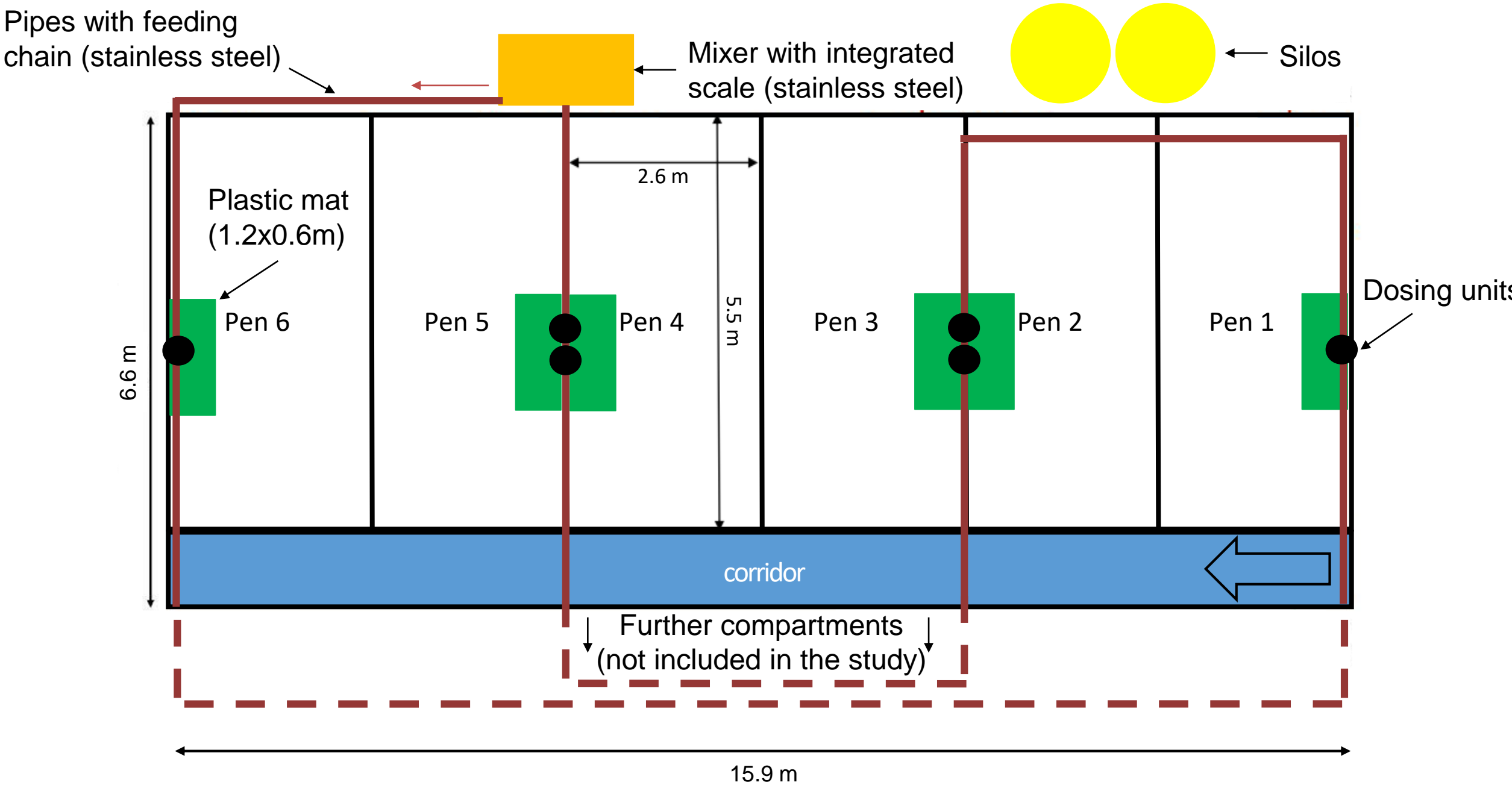

Supplement: S1 File — (PDF) [file pone.0309244.s002.pdf]
