# Supplementary material for: Use of organic material provided by an automatic enrichment device by weaner pigs and its influence on tail lesions
Source: PLoS One. 2024 Nov 1;19(11):e0309244. doi: 10.1371/journal.pone.0309244 (PMC11530003; doi:10.1371/journal.pone.0309244)
Supplement: S2 File — (PDF) [file pone.0309244.s003.pdf]

```
Generalized linear mixed model fit by maximum likelihood (Laplace Approximation) ['glmerMod']
 Family: binomial  ( logit )
Formula: (cbind(DATANEW$Enrichment_Device, DATANEW$Not_Enrichment_Device)) ~ 
    Material * Supplies * Time_Day * I(Scan - mean(Scan)) + I(Week -          mean(Week)) + I(Week - mean(W
eek)):Material + I(Week - 
        mean(Week)):Supplies + I(Week - mean(Week)):Time_Day + 
        I(Week - mean(Week)):Material:Supplies + I(Week - mean(Week)):Material:Time_Day + 
        I(Week - mean(Week)):Supplies:Time_Day + I(Week - mean(Week)):Material:Supplies:Time_Day + 
        I(Week - mean(Week)):I(Scan - mean(Scan)) + (1 | Pen) +          (1 | Batch)
   Data: DATANEW

     AIC      BIC   logLik deviance df.resid 
 64115.0  64536.6 -32000.5  64001.0    11997 

Scaled residuals: 
    Min      1Q  Median      3Q     Max 
-6.1430 -0.9117 -0.0202  0.9228  5.9438 

Random effects:
 Groups Name        Variance Std.Dev.
 Pen    (Intercept) 0.003081 0.05551 
 Batch  (Intercept) 0.011481 0.10715 
Number of obs: 12054, groups:  Pen, 6; Batch, 6

Fixed effects:
                                                            Estimate Std. Error z value Pr(>|z|)    
(Intercept)                                               -0.8670199  0.0832039 -10.420  < 2e-16 ***
MaterialMI                                                -0.0144517  0.0372307  -0.388 0.697892    
MaterialOB                                                 0.0929762  0.0368931   2.520 0.011730 *  
Supplies4                                                 -0.4664545  0.1129221  -4.131 3.62e-05 ***
Supplies6                                                 -0.4781275  0.1115719  -4.285 1.82e-05 ***
Time_DayMorning                                           -0.0515346  0.0359282  -1.434 0.151465    
I(Scan - mean(Scan))                                      -0.1377091  0.0058758 -23.437  < 2e-16 ***
I(Week - mean(Week))                                      -0.1626797  0.0144633 -11.248  < 2e-16 ***
MaterialMI:Supplies4                                       0.0517441  0.0499022   1.037 0.299777    
MaterialOB:Supplies4                                       0.1521246  0.0493705   3.081 0.002061 ** 
MaterialMI:Supplies6                                       0.2931352  0.0443401   6.611 3.82e-11 ***
MaterialOB:Supplies6                                       0.2498304  0.0446132   5.600 2.14e-08 ***
MaterialMI:Time_DayMorning                                -0.1224020  0.0515958  -2.372 0.017677 *  
MaterialOB:Time_DayMorning                                -0.0446171  0.0502570  -0.888 0.374660    
Supplies4:Time_DayMorning                                 -0.2271456  0.0494554  -4.593 4.37e-06 ***
Supplies6:Time_DayMorning                                 -0.2129496  0.0435193  -4.893 9.92e-07 ***
MaterialMI:I(Scan - mean(Scan))                            0.0097492  0.0083005   1.175 0.240181    
MaterialOB:I(Scan - mean(Scan))                            0.0048536  0.0082466   0.589 0.556162    
Supplies4:I(Scan - mean(Scan))                             0.0044395  0.0078981   0.562 0.574049    
Supplies6:I(Scan - mean(Scan))                             0.0047273  0.0069848   0.677 0.498533    
Time_DayMorning:I(Scan - mean(Scan))                       0.0321162  0.0083523   3.845 0.000120 ***
MaterialMI:I(Week - mean(Week))                           -0.1326750  0.0207642  -6.390 1.66e-10 ***
MaterialOB:I(Week - mean(Week))                           -0.0987521  0.0204953  -4.818 1.45e-06 ***
Supplies4:I(Week - mean(Week))                            -0.1507983  0.0197431  -7.638 2.21e-14 ***
Supplies6:I(Week - mean(Week))                            -0.0976494  0.0172824  -5.650 1.60e-08 ***
Time_DayMorning:I(Week - mean(Week))                      -0.0103212  0.0209996  -0.491 0.623077    
I(Scan - mean(Scan)):I(Week - mean(Week))                 -0.0186905  0.0006558 -28.501  < 2e-16 ***
MaterialMI:Supplies4:Time_DayMorning                       0.2411074  0.0688434   3.502 0.000461 ***
MaterialOB:Supplies4:Time_DayMorning                       0.2178681  0.0663608   3.283 0.001027 ** 
MaterialMI:Supplies6:Time_DayMorning                       0.1288145  0.0615574   2.093 0.036386 *  
MaterialOB:Supplies6:Time_DayMorning                       0.1408663  0.0603552   2.334 0.019598 *  
MaterialMI:Supplies4:I(Scan - mean(Scan))                  0.0020434  0.0109708   0.186 0.852242    
MaterialOB:Supplies4:I(Scan - mean(Scan))                 -0.0179908  0.0107093  -1.680 0.092974 .  
MaterialMI:Supplies6:I(Scan - mean(Scan))                 -0.0193416  0.0098073  -1.972 0.048590 *  
MaterialOB:Supplies6:I(Scan - mean(Scan))                 -0.0104903  0.0097598  -1.075 0.282443    
MaterialMI:Time_DayMorning:I(Scan - mean(Scan))           -0.0174228  0.0118588  -1.469 0.141781    
MaterialOB:Time_DayMorning:I(Scan - mean(Scan))            0.0111603  0.0116348   0.959 0.337449    
Supplies4:Time_DayMorning:I(Scan - mean(Scan))             0.0269071  0.0114006   2.360 0.018268 *  
Supplies6:Time_DayMorning:I(Scan - mean(Scan))            -0.0019972  0.0100479  -0.199 0.842447    
MaterialMI:Supplies4:I(Week - mean(Week))                  0.1991675  0.0275106   7.240 4.50e-13 ***
MaterialOB:Supplies4:I(Week - mean(Week))                  0.1279636  0.0267763   4.779 1.76e-06 ***
MaterialMI:Supplies6:I(Week - mean(Week))                  0.1256595  0.0245647   5.115 3.13e-07 ***
MaterialOB:Supplies6:I(Week - mean(Week))                  0.0467462  0.0243665   1.918 0.055053 .  
MaterialMI:Time_DayMorning:I(Week - mean(Week))            0.0287759  0.0299405   0.961 0.336501    
MaterialOB:Time_DayMorning:I(Week - mean(Week))            0.1014659  0.0292779   3.466 0.000529 ***
Supplies4:Time_DayMorning:I(Week - mean(Week))             0.1926154  0.0289788   6.647 3.00e-11 ***
Supplies6:Time_DayMorning:I(Week - mean(Week))             0.0217627  0.0253220   0.859 0.390099    
MaterialMI:Supplies4:Time_DayMorning:I(Scan - mean(Scan)) -0.0478938  0.0156956  -3.051 0.002278 ** 
MaterialOB:Supplies4:Time_DayMorning:I(Scan - mean(Scan)) -0.0293650  0.0152585  -1.924 0.054292 .  
MaterialMI:Supplies6:Time_DayMorning:I(Scan - mean(Scan))  0.0033750  0.0141225   0.239 0.811123    
MaterialOB:Supplies6:Time_DayMorning:I(Scan - mean(Scan)) -0.0168041  0.0139103  -1.208 0.227036    
MaterialMI:Supplies4:Time_DayMorning:I(Week - mean(Week)) -0.2748257  0.0397608  -6.912 4.78e-12 ***
MaterialOB:Supplies4:Time_DayMorning:I(Week - mean(Week)) -0.2449514  0.0385919  -6.347 2.19e-10 ***
MaterialMI:Supplies6:Time_DayMorning:I(Week - mean(Week))  0.0012232  0.0356939   0.034 0.972663    
MaterialOB:Supplies6:Time_DayMorning:I(Week - mean(Week)) -0.1300308  0.0351595  -3.698 0.000217 ***
---
Signif. codes:  0 ‘***’ 0.001 ‘**’ 0.01 ‘*’ 0.05 ‘.’ 0.1 ‘ ’ 1
```
