# Supplementary material for: Use of organic material provided by an automatic enrichment device by weaner pigs and its influence on tail lesions
Source: PLoS One. 2024 Nov 1;19(11):e0309244. doi: 10.1371/journal.pone.0309244 (PMC11530003; doi:10.1371/journal.pone.0309244)
Supplement: S3 File — (PDF) [file pone.0309244.s004.pdf]

```
Analysis of Deviance Table (Type II Wald chisquare tests)

Response: (cbind(DATANEW$Enrichment_Device, DATANEW$Not_Enrichment_Device))
                                                  Chisq Df Pr(>Chisq)    
Material                                       792.3423  2  < 2.2e-16 ***
Supplies                                        16.9874  2  0.0002048 ***
Time_Day                                       590.2504  1  < 2.2e-16 ***
I(Scan - mean(Scan))                         11432.0204  1  < 2.2e-16 ***
I(Week - mean(Week))                          7591.3340  1  < 2.2e-16 ***
Material:Supplies                              165.9411  4  < 2.2e-16 ***
Material:Time_Day                               20.6056  2  3.354e-05 ***
Supplies:Time_Day                               20.9833  2  2.777e-05 ***
Material:I(Scan - mean(Scan))                   24.2496  2  5.423e-06 ***
Supplies:I(Scan - mean(Scan))                   12.3182  2  0.0021142 ** 
Time_Day:I(Scan - mean(Scan))                  152.5088  1  < 2.2e-16 ***
Material:I(Week - mean(Week))                   60.9441  2  5.837e-14 ***
Supplies:I(Week - mean(Week))                   51.2525  2  7.424e-12 ***
Time_Day:I(Week - mean(Week))                   20.6759  1  5.440e-06 ***
I(Scan - mean(Scan)):I(Week - mean(Week))      812.3339  1  < 2.2e-16 ***
Material:Supplies:Time_Day                      44.9762  4  4.021e-09 ***
Material:Supplies:I(Scan - mean(Scan))          19.7263  4  0.0005655 ***
Material:Time_Day:I(Scan - mean(Scan))          34.5890  2  3.084e-08 ***
Supplies:Time_Day:I(Scan - mean(Scan))           2.1950  2  0.3337056    
Material:Supplies:I(Week - mean(Week))          88.1051  4  < 2.2e-16 ***
Material:Time_Day:I(Week - mean(Week))          11.3799  2  0.0033797 ** 
Supplies:Time_Day:I(Week - mean(Week))           5.6931  2  0.0580431 .  
Material:Supplies:Time_Day:I(Scan - mean(Scan))   20.9480  4  0.0003243 ***
Material:Supplies:Time_Day:I(Week - mean(Week))   99.4941  4  < 2.2e-16 ***
---
Signif. codes:  0 ‘***’ 0.001 ‘**’ 0.01 ‘*’ 0.05 ‘.’ 0.1 ‘ ’ 1
```
