# Supplementary material for: Use of organic material provided by an automatic enrichment device by weaner pigs and its influence on tail lesions
Source: PLoS One. 2024 Nov 1;19(11):e0309244. doi: 10.1371/journal.pone.0309244 (PMC11530003; doi:10.1371/journal.pone.0309244)
Supplement: S4 File — (PDF) [file pone.0309244.s005.pdf]

```
Generalized linear mixed model fit by maximum likelihood (Laplace Approximation) ['glmerMod']
 Family: binomial  ( logit )
Formula: tail_lesion_binom2 ~ Material * Supplies * Week + (1 | Pen) +      (1 | Batch)
   Data: TB

     AIC      BIC   logLik deviance df.resid 
  5233.9   5637.7  -2561.0   5121.9     9939 

Scaled residuals: 
    Min      1Q  Median      3Q     Max 
-2.4526 -0.2928 -0.1458 -0.0755 26.1100 

Random effects:
 Groups Name        Variance Std.Dev.
 Pen    (Intercept) 0.4994   0.7067  
 Batch  (Intercept) 0.2074   0.4554  
Number of obs: 9995, groups:  Pen, 6; Batch, 6

Fixed effects:
                             Estimate Std. Error z value Pr(>|z|)    
(Intercept)                 -4.374109   0.745890  -5.864 4.51e-09 ***
MaterialLu                 -11.996813  13.429706  -0.893   0.3717    
MaterialMi                  -0.374865   0.817369  -0.459   0.6465    
Supplies4                   -0.483321   1.308911  -0.369   0.7119    
Supplies6                   -1.661597   1.331728  -1.248   0.2121    
Week2                        1.358094   0.667394   2.035   0.0419 *  
Week3                        1.456598   0.668384   2.179   0.0293 *  
Week4                        2.776153   0.628138   4.420 9.89e-06 ***
Week5                        4.259608   0.616161   6.913 4.74e-12 ***
Week6                        5.217746   0.618826   8.432  < 2e-16 ***
MaterialLu:Supplies4        12.979565  13.400557   0.969   0.3328    
MaterialMi:Supplies4         0.962831   1.435864   0.671   0.5025    
MaterialLu:Supplies6        13.053123  13.624058   0.958   0.3380    
MaterialMi:Supplies6         1.261408   1.806939   0.698   0.4851    
MaterialLu:Week2            11.554650  13.386502   0.863   0.3881    
MaterialMi:Week2            -0.096297   0.919502  -0.105   0.9166    
MaterialLu:Week3            11.944935  13.435838   0.889   0.3740    
MaterialMi:Week3            -0.194588   0.921694  -0.211   0.8328    
MaterialLu:Week4            11.144564  13.460282   0.828   0.4077    
MaterialMi:Week4             0.621584   0.850916   0.730   0.4651    
MaterialLu:Week5            11.524681  13.436062   0.858   0.3910    
MaterialMi:Week5             0.152713   0.838699   0.182   0.8555    
MaterialLu:Week6            11.249053  13.404742   0.839   0.4014    
MaterialMi:Week6            -0.061826   0.841772  -0.073   0.9415    
Supplies4:Week2              0.046469   1.363040   0.034   0.9728    
Supplies6:Week2              0.293395   1.352260   0.217   0.8282    
Supplies4:Week3              0.366085   1.328826   0.275   0.7829    
Supplies6:Week3              0.389132   1.354832   0.287   0.7739    
Supplies4:Week4             -0.953290   1.320513  -0.722   0.4704    
Supplies6:Week4              0.153077   1.282676   0.119   0.9050    
Supplies4:Week5             -0.271843   1.245416  -0.218   0.8272    
Supplies6:Week5              0.038086   1.261177   0.030   0.9759    
Supplies4:Week6             -0.566189   1.245659  -0.455   0.6494    
Supplies6:Week6             -0.869594   1.255052  -0.693   0.4884    
MaterialLu:Supplies4:Week2 -14.609678  13.321738  -1.097   0.2728    
MaterialMi:Supplies4:Week2  -0.888761   1.636600  -0.543   0.5871    
MaterialLu:Supplies6:Week2 -13.205513  13.572050  -0.973   0.3306    
MaterialMi:Supplies6:Week2  -0.440844   1.988257  -0.222   0.8245    
MaterialLu:Supplies4:Week3 -13.767346  13.384744  -1.029   0.3037    
MaterialMi:Supplies4:Week3  -1.397952   1.613156  -0.867   0.3862    
MaterialLu:Supplies6:Week3 -13.789988  13.754027  -1.003   0.3160    
MaterialMi:Supplies6:Week3  -0.242077   1.996210  -0.121   0.9035    
MaterialLu:Supplies4:Week4 -14.617807  13.715096  -1.066   0.2865    
MaterialMi:Supplies4:Week4  -2.025324   1.577363  -1.284   0.1991    
MaterialLu:Supplies6:Week4 -14.775328  13.594080  -1.087   0.2771    
MaterialMi:Supplies6:Week4  -1.911246   1.895737  -1.008   0.3134    
MaterialLu:Supplies4:Week5 -14.562399  13.414767  -1.086   0.2777    
MaterialMi:Supplies4:Week5  -3.419289   1.498772  -2.281   0.0225 *  
MaterialLu:Supplies6:Week5 -12.269721  13.634712  -0.900   0.3682    
MaterialMi:Supplies6:Week5  -0.193355   1.827947  -0.106   0.9158    
MaterialLu:Supplies4:Week6 -13.965931  13.369838  -1.045   0.2962    
MaterialMi:Supplies4:Week6  -2.809261   1.475651  -1.904   0.0569 .  
MaterialLu:Supplies6:Week6 -11.943984  13.576293  -0.880   0.3790    
MaterialMi:Supplies6:Week6  -0.006115   1.810669  -0.003   0.9973    
---
Signif. codes:  0 ‘***’ 0.001 ‘**’ 0.01 ‘*’ 0.05 ‘.’ 0.1 ‘ ’ 1
```
