# Supplementary material for: Use of organic material provided by an automatic enrichment device by weaner pigs and its influence on tail lesions
Source: PLoS One. 2024 Nov 1;19(11):e0309244. doi: 10.1371/journal.pone.0309244 (PMC11530003; doi:10.1371/journal.pone.0309244)
Supplement: S5 File — (PDF) [file pone.0309244.s006.pdf]

```
Analysis of Deviance Table (Type II Wald chisquare tests)

Response: tail_lesion_binom2
                          Chisq Df Pr(>Chisq)    
Material                 48.626  2  2.761e-11 ***
Supplies                 11.901  2   0.002604 ** 
Week                    920.238  5  < 2.2e-16 ***
Material:Supplies        75.266  4  1.751e-15 ***
Material:Week            15.152 10   0.126621    
Supplies:Week            58.555 10  6.794e-09 ***
Material:Supplies:Week   36.795 20   0.012381 *  
---
Signif. codes:  0 ‘***’ 0.001 ‘**’ 0.01 ‘*’ 0.05 ‘.’ 0.1 ‘ ’ 1
```
