# Supplementary material for: Use of organic material provided by an automatic enrichment device by weaner pigs and its influence on tail lesions
Source: PLoS One. 2024 Nov 1;19(11):e0309244. doi: 10.1371/journal.pone.0309244 (PMC11530003; doi:10.1371/journal.pone.0309244)
Supplement: S6 File — (PDF) [file pone.0309244.s007.pdf]

```
Generalized linear mixed model fit by maximum likelihood (Laplace Approximation) ['glmerMod']
 Family: binomial  ( logit )
Formula: tail_length_loss_binom ~ Supplies * Material + (1 | Pen) + (1 |      Batch)
   Data: TL

     AIC      BIC   logLik deviance df.resid 
   559.9    611.6   -269.0    537.9      803 

Scaled residuals: 
    Min      1Q  Median      3Q     Max 
-2.5298 -0.3254 -0.1441 -0.0281  8.5236 

Random effects:
 Groups Name        Variance Std.Dev.
 Pen    (Intercept) 1.609    1.269   
 Batch  (Intercept) 2.942    1.715   
Number of obs: 814, groups:  Pen, 6; Batch, 6

Fixed effects:
                     Estimate Std. Error z value Pr(>|z|)   
(Intercept)          -0.06021    1.36377  -0.044  0.96478   
Supplies4            -1.89535    1.88603  -1.005  0.31492   
Supplies6            -3.34839    1.83535  -1.824  0.06809 . 
MaterialLu           -1.52698    0.49786  -3.067  0.00216 **
MaterialMi           -0.59647    0.54848  -1.088  0.27681   
Supplies4:MaterialLu -3.10972    0.95824  -3.245  0.00117 **
Supplies6:MaterialLu  2.91287    0.88534   3.290  0.00100 **
Supplies4:MaterialMi -2.98276    1.11810  -2.668  0.00764 **
Supplies6:MaterialMi  0.42831    1.00761   0.425  0.67078   
---
Signif. codes:  0 ‘***’ 0.001 ‘**’ 0.01 ‘*’ 0.05 ‘.’ 0.1 ‘ ’ 1
```
