# Supplementary material for: Use of organic material provided by an automatic enrichment device by weaner pigs and its influence on tail lesions
Source: PLoS One. 2024 Nov 1;19(11):e0309244. doi: 10.1371/journal.pone.0309244 (PMC11530003; doi:10.1371/journal.pone.0309244)
Supplement: S7 File — (PDF) [file pone.0309244.s008.pdf]

```
Analysis of Deviance Table (Type II Wald chisquare tests)

Response: tail_length_loss_binom
                    Chisq Df Pr(>Chisq)
Supplies           2.1731  2   0.337377
Material          13.5729  2   0.001129 **
Supplies:Material 48.4295  4  7.679e-10 ***
---
Signif. codes:  0 ‘***’ 0.001 ‘**’ 0.01 ‘*’ 0.05 ‘.’ 0.1 ‘ ’ 1
```
