# Supplementary material for: Use of organic material provided by an automatic enrichment device by weaner pigs and its influence on tail lesions
Source: PLoS One. 2024 Nov 1;19(11):e0309244. doi: 10.1371/journal.pone.0309244 (PMC11530003; doi:10.1371/journal.pone.0309244)
Supplement: S8 File — (PDF) [file pone.0309244.s009.pdf]

```
Linear mixed model fit by REML ['lmerMod']
Formula: ADG_rearing_total ~ Supplies * Material + (1 | Batch) + (1 |      Pen)
   Data: Tier

REML criterion at convergence: 9354.6

Scaled residuals: 
    Min      1Q  Median      3Q     Max 
-3.2110 -0.6644 -0.0032  0.6104  3.1857 

Random effects:
 Groups   Name        Variance Std.Dev.
 Batch    (Intercept)  359.82  18.97   
 Pen      (Intercept)   10.17   3.19   
 Residual             6511.44  80.69   
Number of obs: 810, groups:  Batch, 6; Pen, 6

Fixed effects:
                     Estimate Std. Error t value
(Intercept)          438.4004    15.9672  27.456
Supplies4             27.3941    23.0007   1.191
Supplies6              8.6381    22.4137   0.385
MaterialMI            40.6099    12.2802   3.307
MaterialOB            31.4730    12.3210   2.554
Supplies4:MaterialMI -56.9724    17.8277  -3.196
Supplies6:MaterialMI -20.7520    17.1317  -1.211
Supplies4:MaterialOB -42.1565    17.9658  -2.346
Supplies6:MaterialOB  -0.8826    17.1883  -0.051
```
