# Supplementary material for: Use of organic material provided by an automatic enrichment device by weaner pigs and its influence on tail lesions
Source: PLoS One. 2024 Nov 1;19(11):e0309244. doi: 10.1371/journal.pone.0309244 (PMC11530003; doi:10.1371/journal.pone.0309244)
Supplement: S9 File — (PDF) [file pone.0309244.s010.pdf]

```
Analysis of Deviance Table (Type II Wald chisquare tests)

Response: ADG_rearing_total
                    Chisq Df Pr(>Chisq)  
Supplies           0.2318  2    0.89057  
Material           8.2071  2    0.01651 *
Supplies:Material 12.3098  4    0.01519 *
---
Signif. codes:  0 ‘***’ 0.001 ‘**’ 0.01 ‘*’ 0.05 ‘.’ 0.1 ‘ ’ 1
```
